# Flapless Localised Management of Sinus Floor (LMSF) for trans-crestal sinus floor augmentation and simultaneous implant placement. A retrospective non-randomized study: 5-year of follow-up

**DOI:** 10.1016/j.heliyon.2021.e07927

**Published:** 2021-09-04

**Authors:** Giovanni Battista Bruschi, Ernesto Bruschi, Laura Papetti

**Affiliations:** Private Practice, Frosinone/Rome, Italy

**Keywords:** Implant, Sinus, Lift, Flapless, Dental Implants, Surgery, Maxillary, Edentulous, Osteotome, Magnetic mallet

## Abstract

**Background:**

Trans-crestal sinus lift procedures are well established.

**Purpose:**

to retrospectively analyse the efficacy of a flapless trans-crestal maxillary sinus floor elevation and simultaneous dental implant placement based on the Localised Management of Sinus Floor (LMSF) technique suitable for cases with sufficient width of keratinized tissue and of crestal bone but insufficient vertical dimensions of the bone below the sinus.

**Materials and methods:**

71 sinus elevations with simultaneous implant placement were performed on 52 consecutive patients. Following an initial pilot bur transmucosal perforation, the Magnetic Mallet was used with progressively larger osteotomes. The mallet osteotomes are initially directed palatally, towards the cortex of the medial wall of the sinus, below the palato-nasal recess (PNR) and then redirected in a more vertical direction to create the final osteotomy for implant placement.

**Results:**

No significant complications were reported in the post-operative phase. The cumulative success rate during the observation period was 95%. All successful implants were successfully loaded with metal-ceramic or monolithic zirconia crowns and bridges and remained in function during the observation period.

**Conclusions:**

Flapless Localised Management of Sinus Floor (LMSF) is a safe and effective surgical technique with minimal risks and with the advantage of low morbidity. Also, only native bone is used for augmentation and there is no need for additional grafting.

## Teaser

Implant; Sinus; Lift; Flapless; Dental; Surgery; Maxillary; Edentulous; Osteotome; Magnetic mallet.

## 1 Introduction

Vertical bone atrophy of edentulous posterior maxilla is due to various factors. Periodontal diseases, non-conservative extractions and preexisting pneumatization are among these. It is a frequent clinical condition. Many different techniques exist for implant insertions in such deficient sites. The classical solution is the lateral window sinus graft [1, 2].

This last surgical technique originates from the original Caldwell-Luc sinus operation. The lateral window sinus graft precedes or is concomitant to implant insertion. Many systematic reviews and meta-analysis prove the efficacy of this treatment [3, 4, 5, 6, 7].

In an attempt to reduce invasiveness and morbidity alternative surgical approaches were born. These comprise the trans-alveolar approaches to vertical augmentation.

Summers recommended osteotome-mediated sinus floor elevation [8, 9]. The Summers technique with immediate implant insertions has two variations. The Osteotome Sinus Floor Elevation (OSFE) is without grafting. The Bone Added Osteotome Sinus Floor Elevation (BAOSFE) prescribes bone grafting below the Schneiderian membrane. The direction of the osteotomes is always straight along the axis of the final osteotomy. Implant insertion is immediate in both variations of the Summer's technique.

Various successive hydraulic, piezoelectric and bone-condensing-bur trans-alveolar techniques obtain similar results [10, 11].

Bruschi et al. proposed a different osteotome technique denominated Localised Management of Sinus Floor (LMSF) [12]. LMSF uses a paramarginal partial-thickness flap to expose the crest. The initial direction of the osteotomes is lateral (medial), towards the palato-nasal recess (PNR) [13]. Sinus elevation starts with a greenstick fracture immediately below the cortex at the medial wall of the sinus. Then osteotome direction becomes more axial while bone shifts at the apex to complete the final sinus elevation and osteotomy. Another major difference is that bone graft material is never used in this technique. Also, Localised Management of Sinus Floor (LMSF) and Edentulous Ridge Expansion (ERE) [14, 15, 16] are combinable for ridge width augmentation. Successive studies confirm the reliability and adaptability of Localised Management of Sinus Floor (LMSF) [17, 18].

Localised Management of Sinus Floor (LMSF) is applicable also for immediate implants in maxillary molar sockets [19]. Recently, a convenient electrical mallet (Magnetic Mallet) replaced the manual mallet [20]. The purpose of the present paper is to study the efficacy of a mini-invasive, flapless modification of the original Localised Management of Sinus Floor (LMSF) technique by following the therapeutic outcome of consecutively treated clinical cases. This technique is suitable only for sites of the posterior maxilla that show insufficient bone below the sinus combined with physiological, normal width of the keratinized tissue and alveolar bone. The hypothesis is that this modified surgical technique, when used for these carefully selected implant sites, is highly reliable and with low morbidity.

The practical purpose of this study is to introduce a surgical variant of classical LMSF technique with a lower mobility that can be adopted for select clinical cases.

## 2 Materials and methods

52 consecutive patients who received, between January 2014 and December 2018, 71 sinus elevations with simultaneous implant placement were selected in 2 privates periodontal practice in Rome, Italy, for a retrospective analysis of the reliable Localised Management of Sinus Floor (LMSF) technique in carefully selected implant site, like upper bicuspid and upper molar areas falling in Kennedy Classes I, II and III.

Patient Selection.

Patients with at least 2 years of follow-up were selected according to the following inclusion criteria:

- Good oral hygiene with full mouth plaque score (FMPS) and full mouth bleeding score (FMBS) at 15% cut-off
- Presence of final restoration such as Single crowns (SCs) and FPDs.

Exclusion criteria included:

- Incomplete medical records
- Sever kidney and liver diseases
- Immunodeficiency states
- History of radiotherapy in the head/neck region
- Poorly controlled diabetes
- oral lesion in the surgery site region
- smoking more 10 cigarettes per day

The study population consisted of patients rehabilitated with metal-ceramic or monolithic zirconia fixed partial dentures (FPDs) and single crowns (Scs). The treatment was performed by two experienced surgeons and the same technician. All investigation reported were carried out in accordance with the 1975 Helsinki Declaration, as revised in 2013 for ethical approval. All participants provided written informed consent after receiving explanations of study objectives and procedures [21].

Because of the retrospective nature of the present study, the institutional review board of the District Medical Committee (Ordine dei Medici di Frosinone) provided written approval with Protocol 0000625/2021/opdmcfr/FR/P.

### 2.1 Surgical technique

All sites had an adequate band of keratinized tissue with no need for augmentation. The keratinized tissue extended 4–5 mm apical to the planned site. Additionally, the cone-beam computed tomography (CBCT) scan showed a large enough width of the alveolar bone combined with insufficient height for the planned implant osteotomy, but with at least 4 mm of native bone below the sinus.

Patients started oral Amoxicillin 875 mg/Clavulanic Acid 125mg (Augmentin 1000 mg, GlaxoSmithKline S.p.A., Via A. Fleming 2, Verona (VR), Italy) on the morning of the surgery and continued thereafter twice a day for a total of 3 days. The surgeons administered local anaesthetic (Articaine hydrochloride 4% with adrenaline 1:100,000 [Septanest, Septodont, Saint-Maur-des-Fossés, France]).

The surgeons inserted two types of root-form bone-level implants during this study: Global UXR with ZrTi surface (Sweden & Martina SpA, Via Veneto 10 - 35020 Due Carrare, Padova, Italy) and Straumann Bone Level Tapered (BLT) titanium with SLA surface (Straumann Holding AG, Peter Merian-Weg 12, 4002 Basel, Switzerland).

The surgery started with a mid-crestal initial osteotomy with lanceolate and/or 1.2 mm round burs. The depth did not exceed 2 mm below the sinus. The resistance to this initial drilling confirmed or not the radiographic bone quality assessment. After the initial preparation, the surgeons expanded the implant osteotomy with the osteotomes. These were progressively larger osteotomes in 0.5 mm increments, mounted on the magnetic mallet (Osseotouch, Osnrgy s.r.l, Piazza Garibaldi, 921013 Gallarate (VA), Italy).

The magnetic mallet is an electromagnetically-driven handpiece that mounts the osteotomes on its tip and delivers a force of up to 90 DaN/8μs [20].

**Figure 1:**
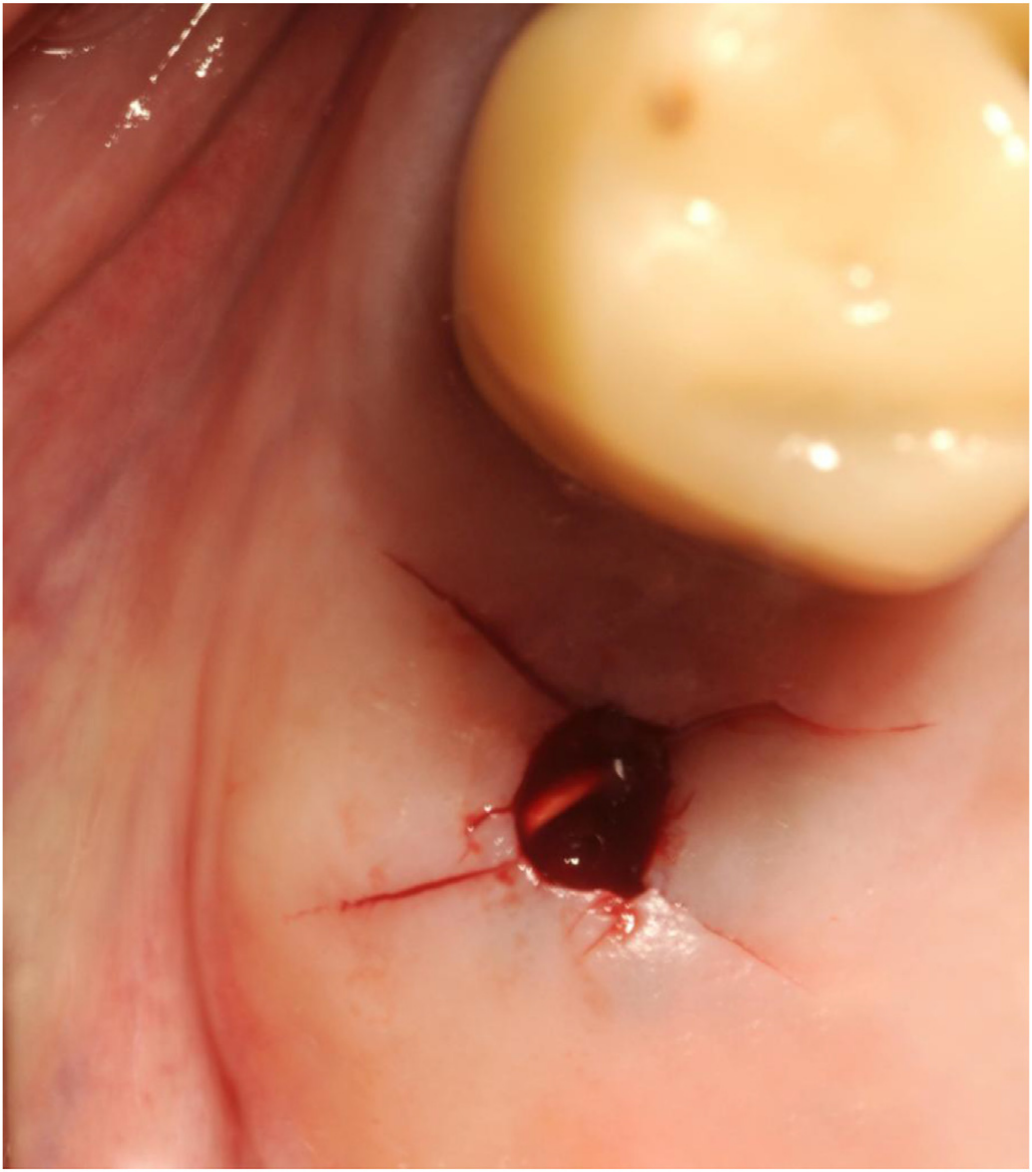
Initial flapless osteotomy with X-shaped mucosal release across the access that is performed in cases where tension builds up in the mucosa during the flapless procedure. The gutta-percha tip is useful to check depth and direction on the successive radiograph.

When the surgeons judged the mucosal tension around the osteotomy to be excessive, they relieved it with an X-shaped incision across the access tunnel (Figure 1).

**Figure 2:**
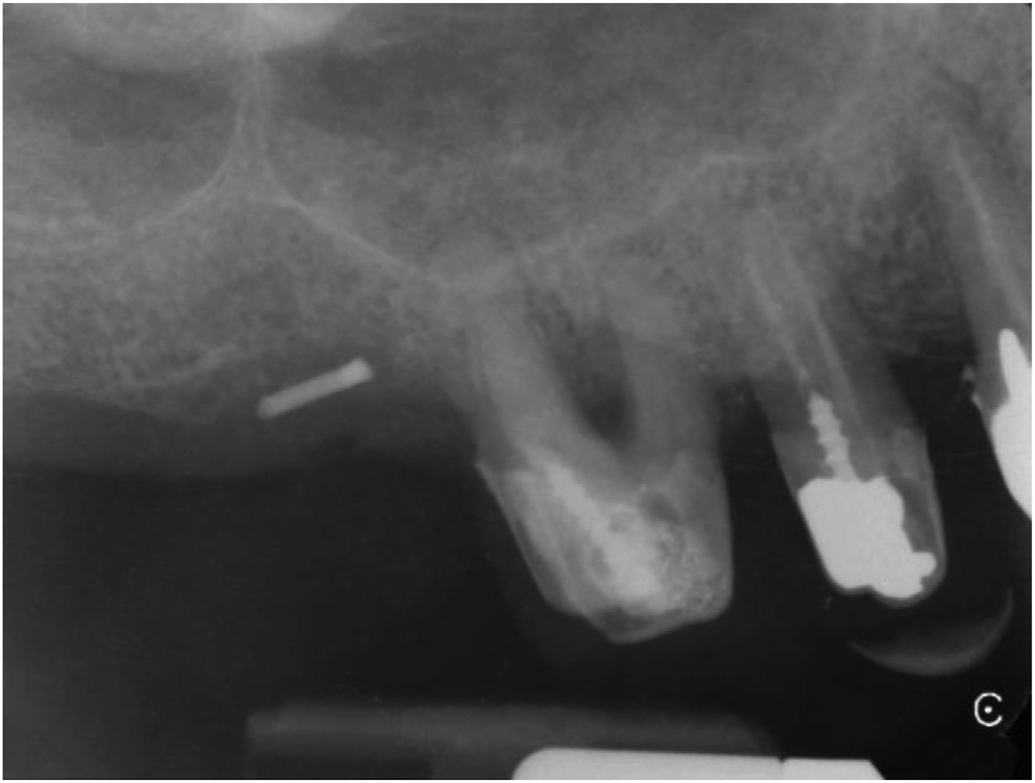
Periapical X-ray taken with the paralleling technique of the clinical situation shown in Figure 1. The gutta-percha tip shows the position of the initial osteotomy with respect to the sinus floor.

The osteotomes engaged the initial bur path. In the beginning, the surgeons directed them at an angle of 30–45° towards the palato-nasal recess (PNR) [13]. The target area is the cortex immediately below the medial wall of the sinus (Figure 2). Here sinus elevation starts with a greenstick fracture. Care is necessary to avoid tearing the Schneiderian membrane.

After the initial slanted osteotomy, the operators applied pressure towards the palate. With this technique, the osteotome-mallet assembly becomes a class 1 lever with the fulcrum on the extremely resistant cortex on the palatal side. The implant site is thus verticalized while the osteotome path becomes progressively larger and more axial. Note that in cases where the alveolar bone crest towards the PNR is thinner, the initial osteotome path must be changed to engage the thicker part of the alveolar bone (as visualised on the preoperative CBCT). This thicker part could be distal, mesial or buccal rather than the usual palatal. In all cases, after the initial elevation, the clinicians directed the osteotomes progressively more axially as described above. There are straight and angled mallet osteotomes that adapt to all clinical situations.

Depth and direction of the osteotomy was checked radiographically when it reached a size of about 2 × 10 mm.

**Figure 3:**
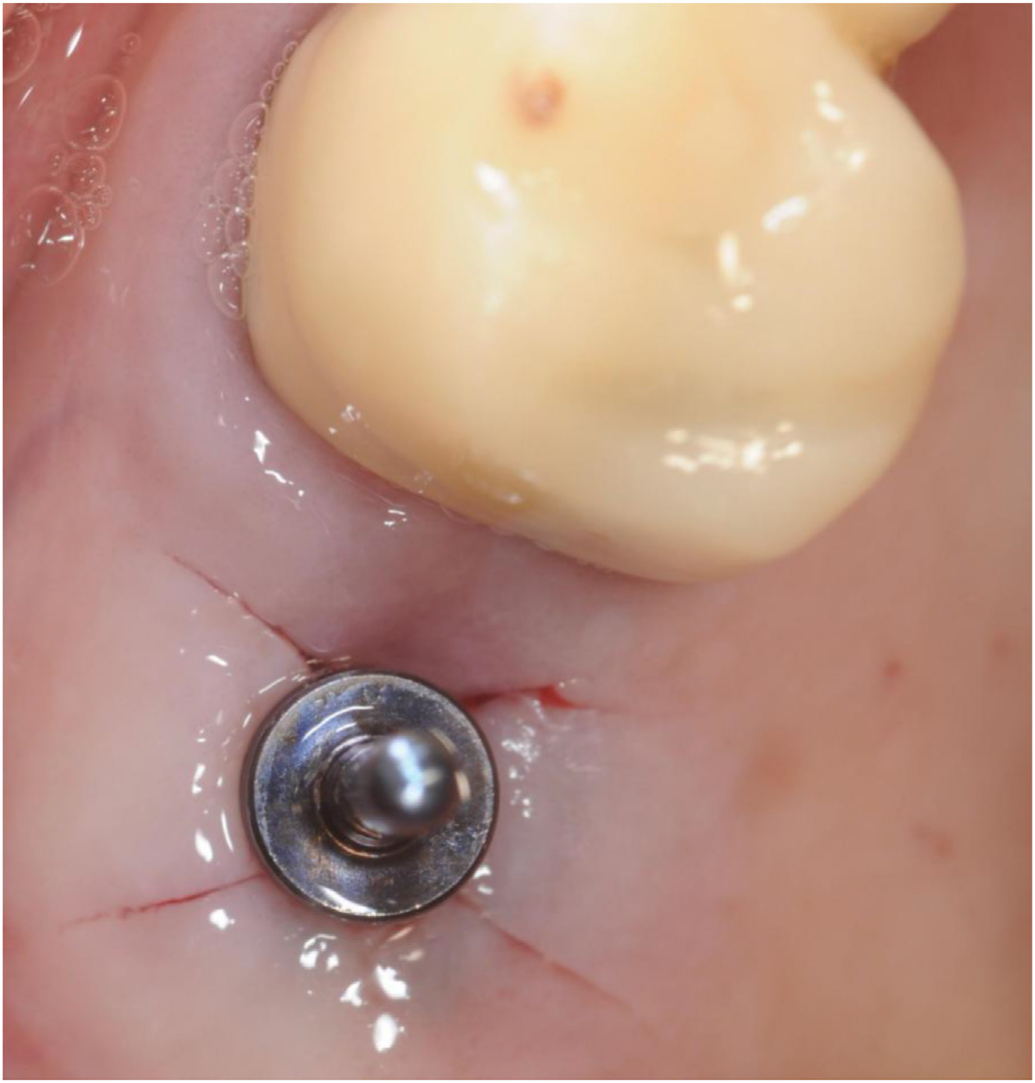
A 10 mm parallel pin in position in the initial osteotomy created with the 2 mm osteotome of the magnetic mallet. The surgeon taps the parallel pin in position and verticalizes it with a special concave-tip insert for the magnetic mallet.

**Figure 4:**
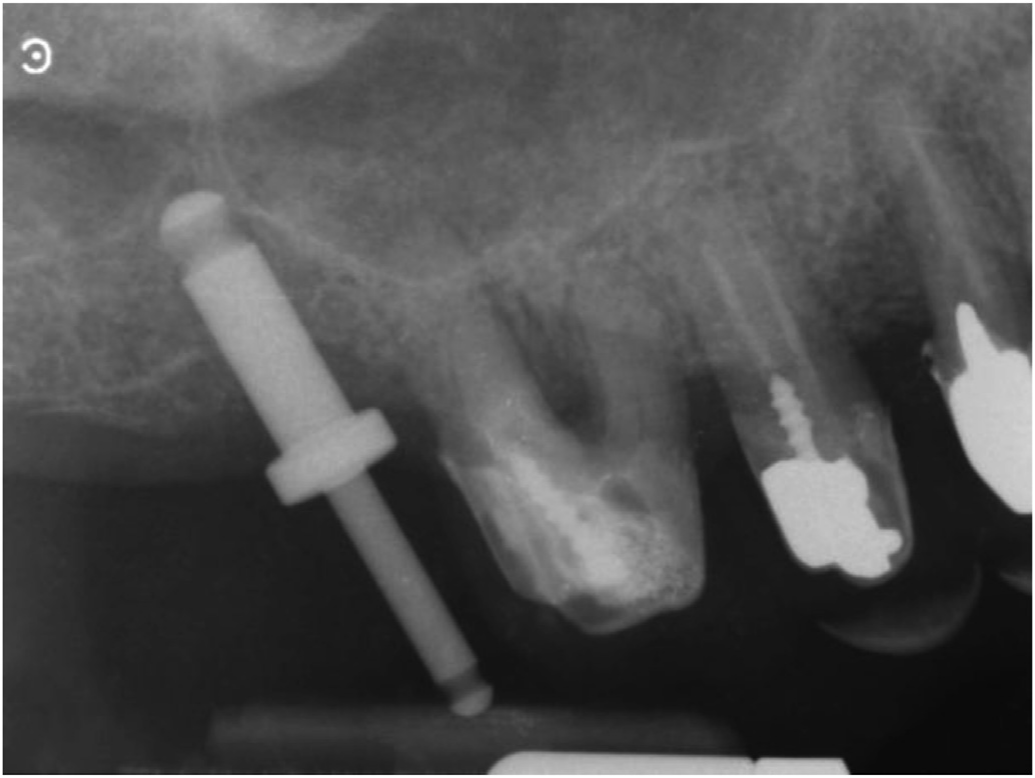
Periapical X-ray taken with the paralleling technique of the clinical situation shown in figure 4. The tip of the 10 mm parallel pin is at the level of the sinus floor cortical bone after tapping it with a special concave-tip insert for the magnetic mallet. All anatomical landmarks are precisely identified and mentally mapped on the surgical field. At this point, the surgeon finalizes the osteotomy with progressively larger magnetic mallet osteotomes in 0.5 mm increments. The osteotome tip is always directed towards the area with more bone (usually palatal towards the PNR) and then verticalized during the successive movements to expand internally the bone above the osteotomy, which extends vertically. The last osteotome corresponds to the diameter of the planned implant (but the osteotome diameter is 0,2-1 mm smaller than the implant diameter to improve stability).

At this stage, a periapical digital X-ray with the paralleling technique was taken with a 10 mm parallel pin. After this check, the surgeons made any necessary corrections to the osteotomy and finalized implant insertion (Figures 3 and 4).

The final osteotomy preparation is 2 mm shorter and with a reduced diameter (0.2–1 mm depending on bone quality) with respect to the planned implant. This is necessary to maximize implant stability.

Before implant insertion, washing the osteotomy with sterile saline removes any detached fragments. A delicate Valsalva manoeuvre confirms the integrity of the Schneiderian membrane.

**Figure 5:**
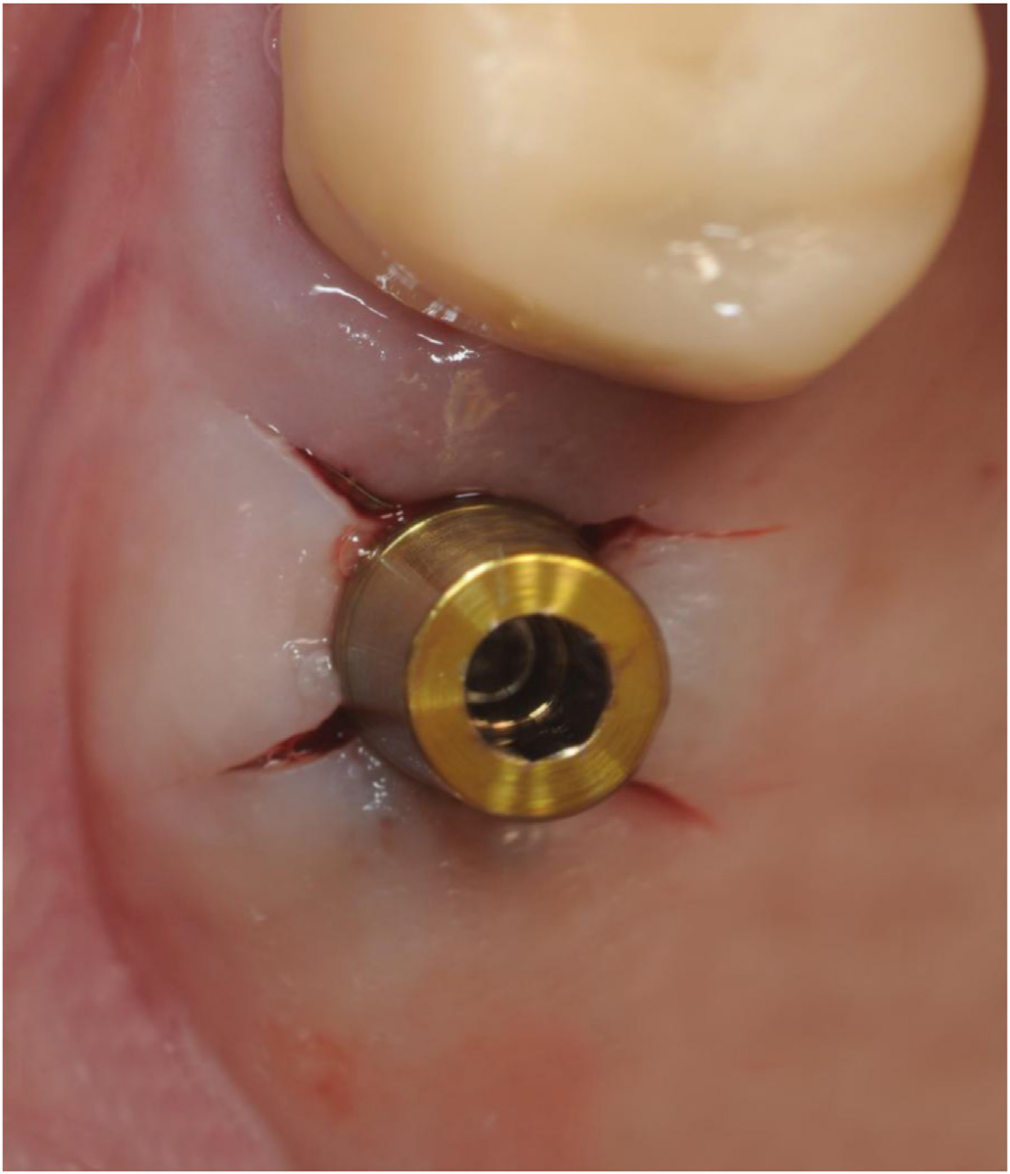
Clinical view of the final implant position immediately after insertion with the gold-coloured titanium driver in place. The implant is a Sweden&Martina Global UXR 5.5 × 13 mm. The final osteotomy preparation is 2 mm shorter and with a reduced diameter with respect to the implant itself. The final sinus lift is obtained during implant insertion. The osteotomy is washed with sterile saline and the implant is inserted with the contra-angle. The final position is achieved with a calibrated manual wrench and additionally corrected, if necessary, by tapping directly with the concave-tip insert of the magnetic mallet directly on the implant driver.

For implant insertion, the contra-angle is set at 15 rpm and 25 Ncm without saline irrigation. The use of a calibrated manual wrench can help to secure the final implant position. A final torque of at least 25–30 Ncm must be obtained (Figure 5).

**Figure 6:**
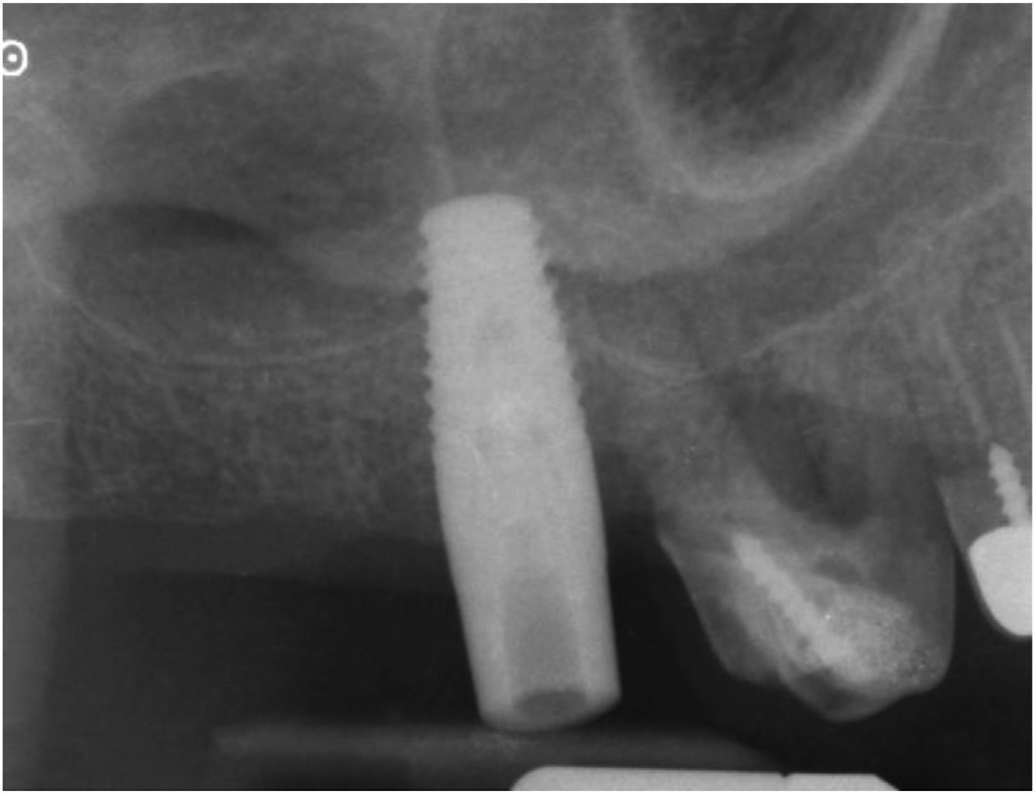
Periapical X-ray taken with the paralleling technique of the clinical situation shown in figure 6, immediately after implant insertion. When compared with Figure 5, it is clear that the implant tip is above the original sinus floor and that is surrounded by some shifted bone structure.

A periapical x-ray taken with the paralleling technique confirms that the implant is in the ideal position (Figure 6). If necessary, the position is corrected by tapping directly with the magnetic mallet and a specific insert on the implant driver. In this technique, the bone elasticity allows fine adjustments of the implant position both vertically and axially.

This is the case with all bone-expanding techniques. The Sweden & Martina Global UXR implant driver proved more suitable for these correction manoeuvres than the “Loxim” component of the Straumann BLT implant, that is designed to detach with excess force.

**Figure 7:**
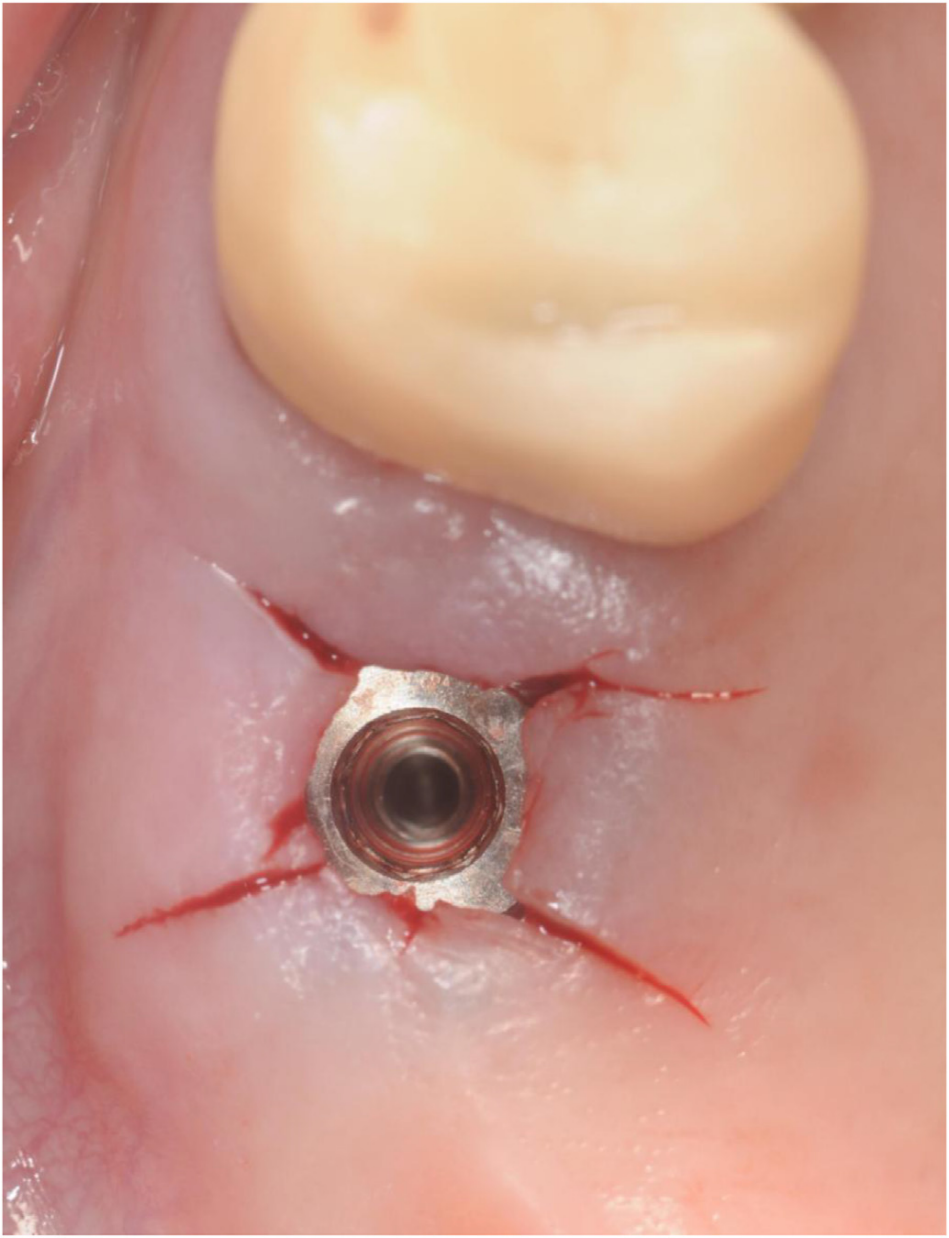
The surgeon removes the implant driver and rinses the implant connection with a 2% chlorhexidine solution.

**Figure 8:**
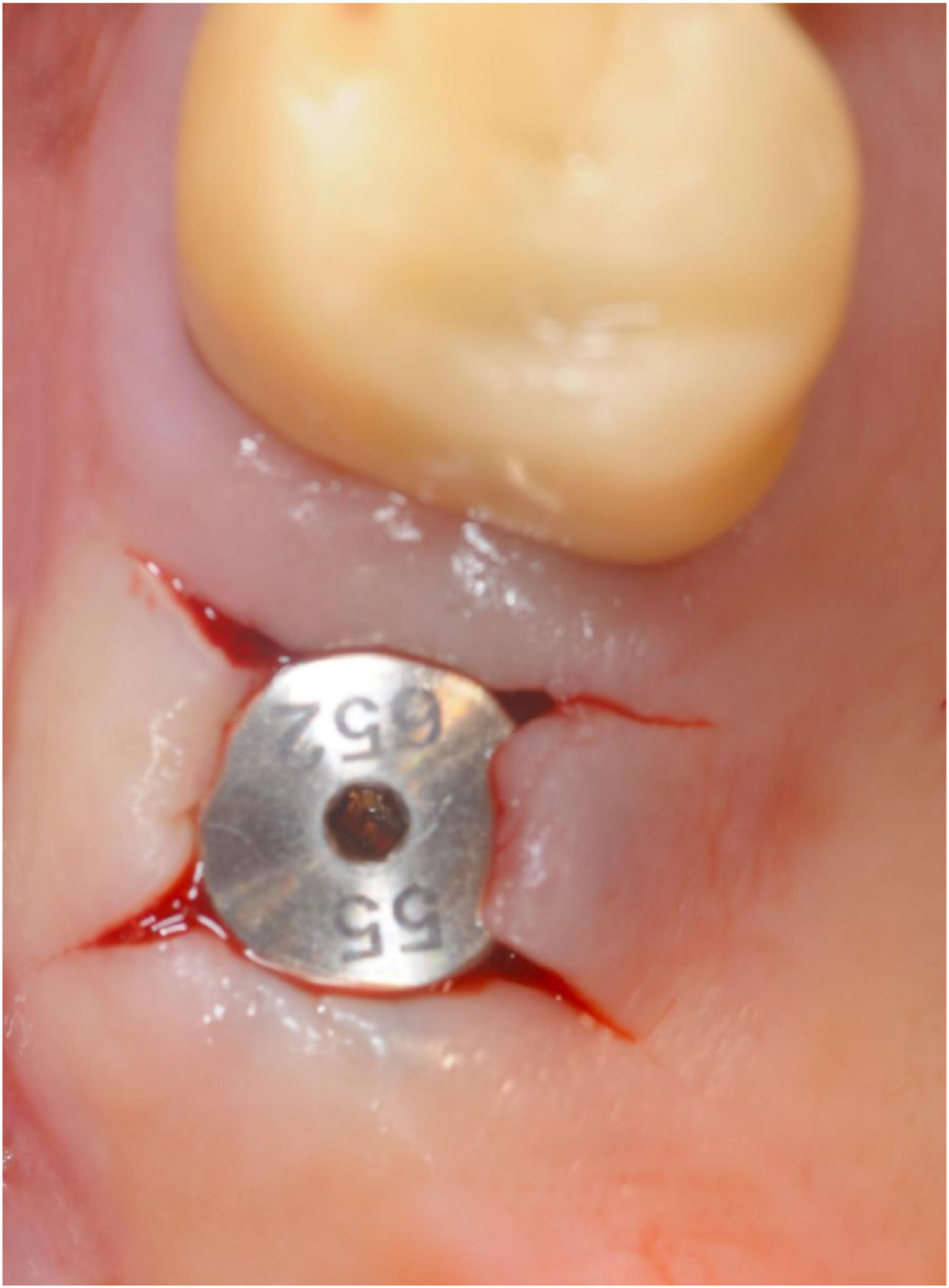
The operator manually tightens a 2 mm sterile healing screw on the implant and instructs the patient to clean the healing screw twice daily with a cotton swab soaked in a 0.05% chlorhexidine.

At this point, the surgeons removed the implant driver. They washed the implant connection with a 2% chlorexidine solution and inserted a 2 mm healing screw (Figures 7 and 8).

They instructed the patients to clean the healing screw twice daily with a cotton swab soaked in a 0.05% chlorexidine (Curasept ADS 0.05%, Curaden Healthcare S.p.A. - Saronno (VA), Italy) mouthwash and dismissed them after a short recovery period.

### 2.2 Follow-up

The operators checked the patients clinically one week after surgery. They performed an X-ray check with the paralleling technique after 5 weeks. If the X-ray check showed normal ossification a prosthetic impression appointment was scheduled immediately thereafter.

**Figure 9:**
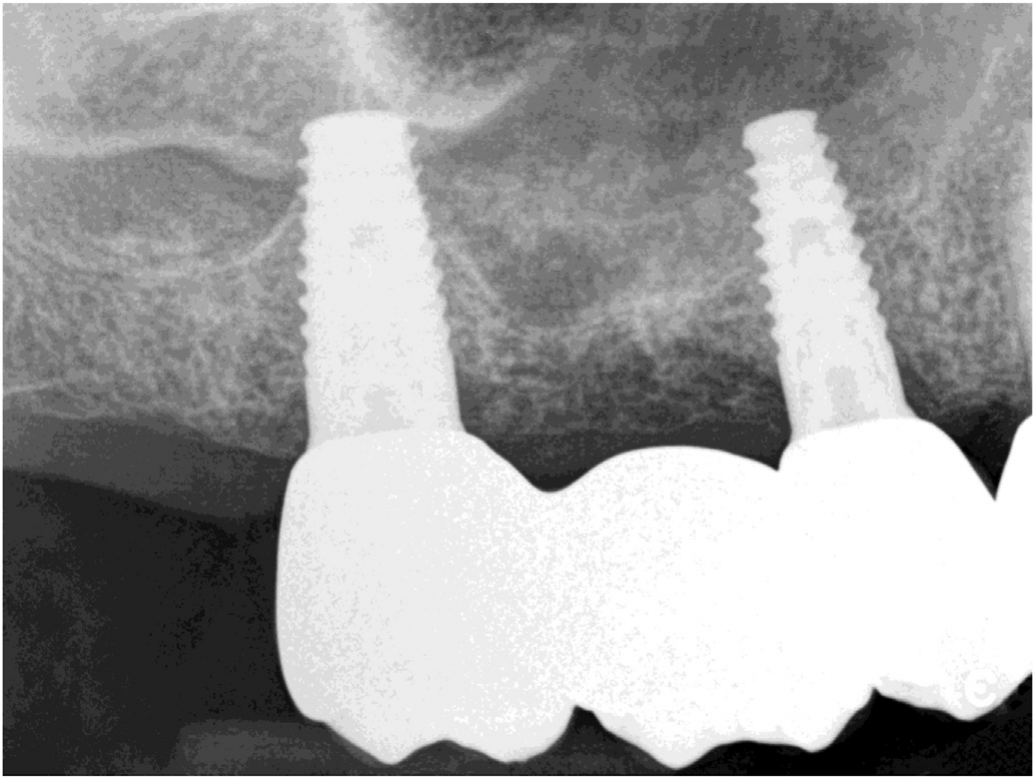
Periapical check X-ray taken with the paralleling technique one year after surgery with the final fixed prosthesis. Ongoing ossification of the elevated bone around the implant apex is evident. The implant in the bicuspid position was inserted with the traditional LMSF technique. The image shows a new elevated line of cortical bone forming between the two implant tips.

A periapical check X-ray taken with the paralleling technique is scheduled after 1 year (Figure 9).

**Figure 10:**
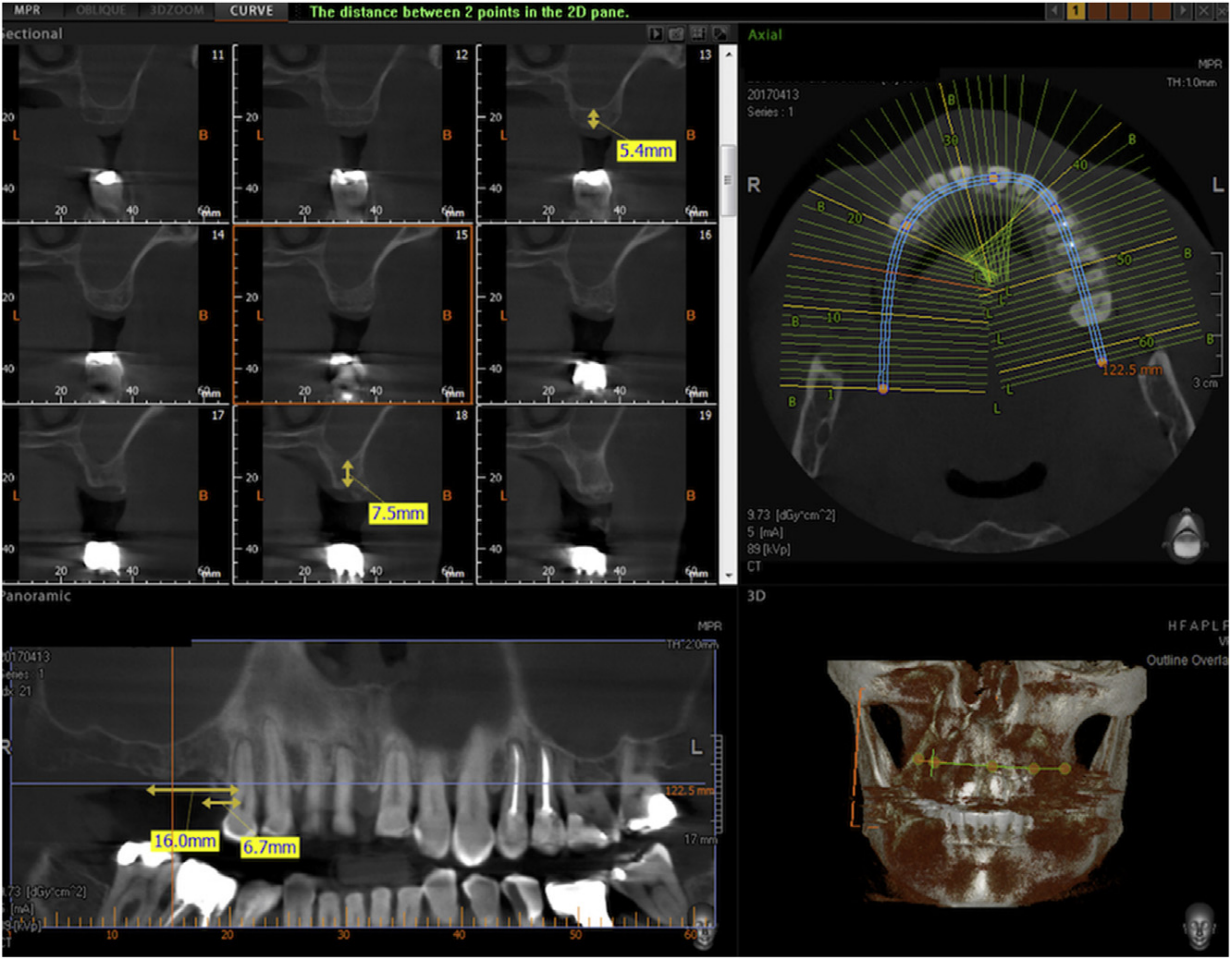
Preoperative CBCT of a case selected for the Flapless LMSF Technique. The treatment plans is to insert 2 implants in the upper right sextant in the positions of the second bicuspid and the first molar. The alveolar bone height is 5.4 mm in the molar site and 7.5 mm in the bicuspid site. The width is sufficient for implant placement without expansion. The CBCT shows signs of chronic sinusitis (asymptomatic). The middle meatus is unobstructed.

**Figure 11:**
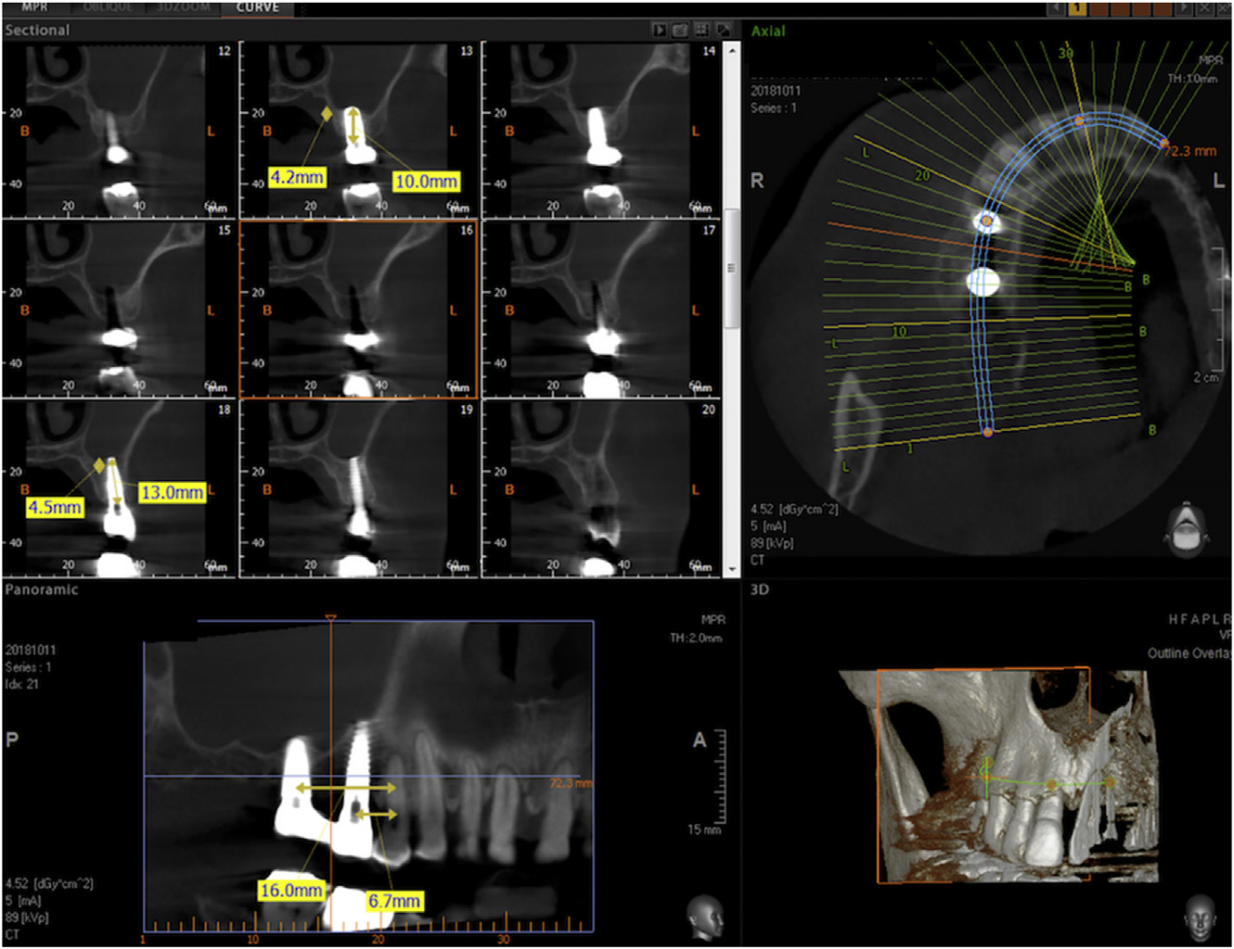
Sectional CBCT of the Flapless LMSF case shown in figure 11 taken one year after surgery and 10 months after prosthetic loading. Both sites show evident vertical bone gain below the Schneiderian membrane. The implant in the first molar position is 4.8 × 10 mm and the implant in the second bicuspid position is 3.8 × 13 mm. Both are Sweden&Martina Global UXR.

**Figure 12:**
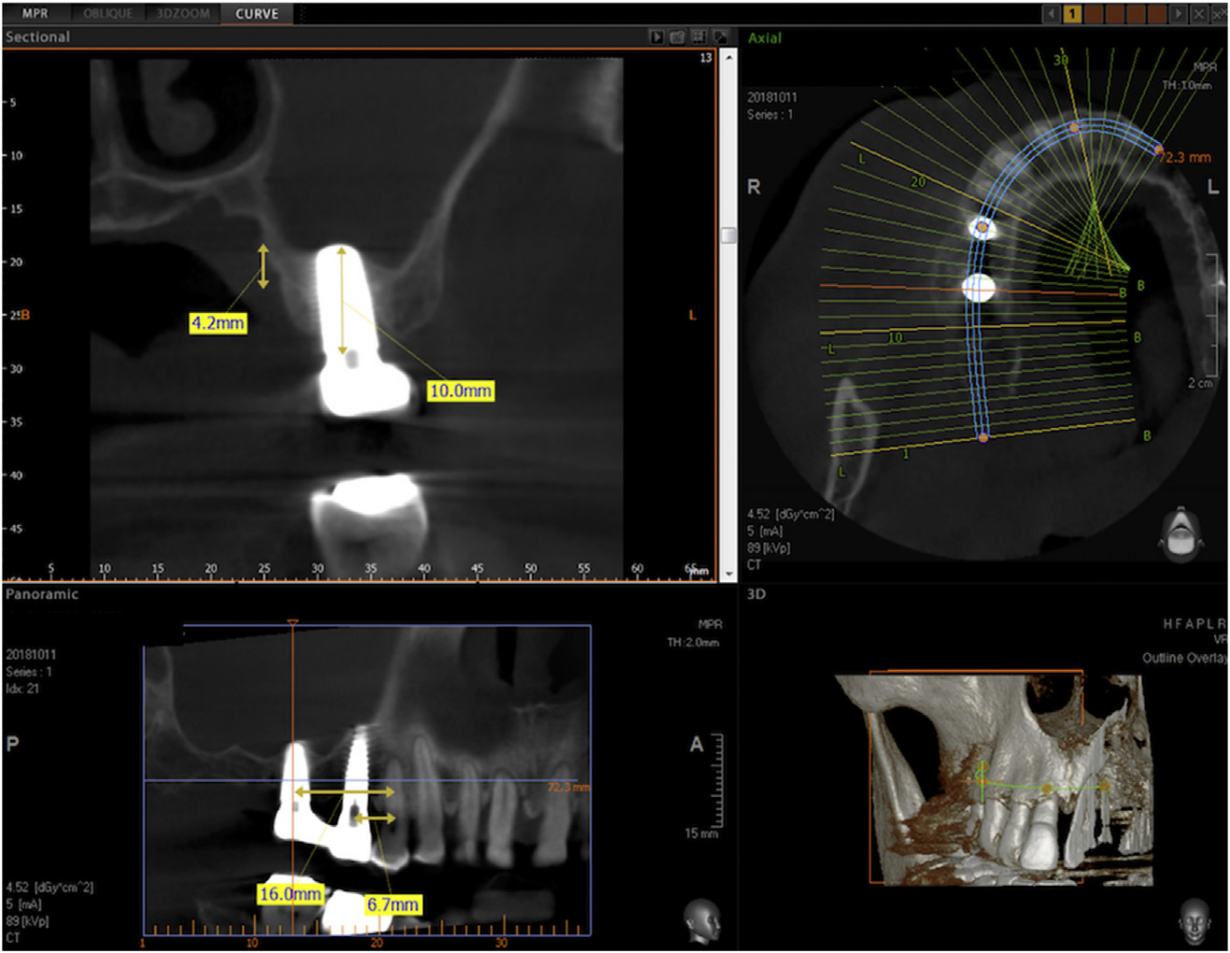
Detail of the CBCT of figure 12 centered on the implant in the first molar site. The implant is a 4.8 × 13 mm Sweden & Martina Global UXR. The apex of the implant is covered with newly formed bone. The vertical bone augmentation is about 4.2 mm.

**Figure 13:**
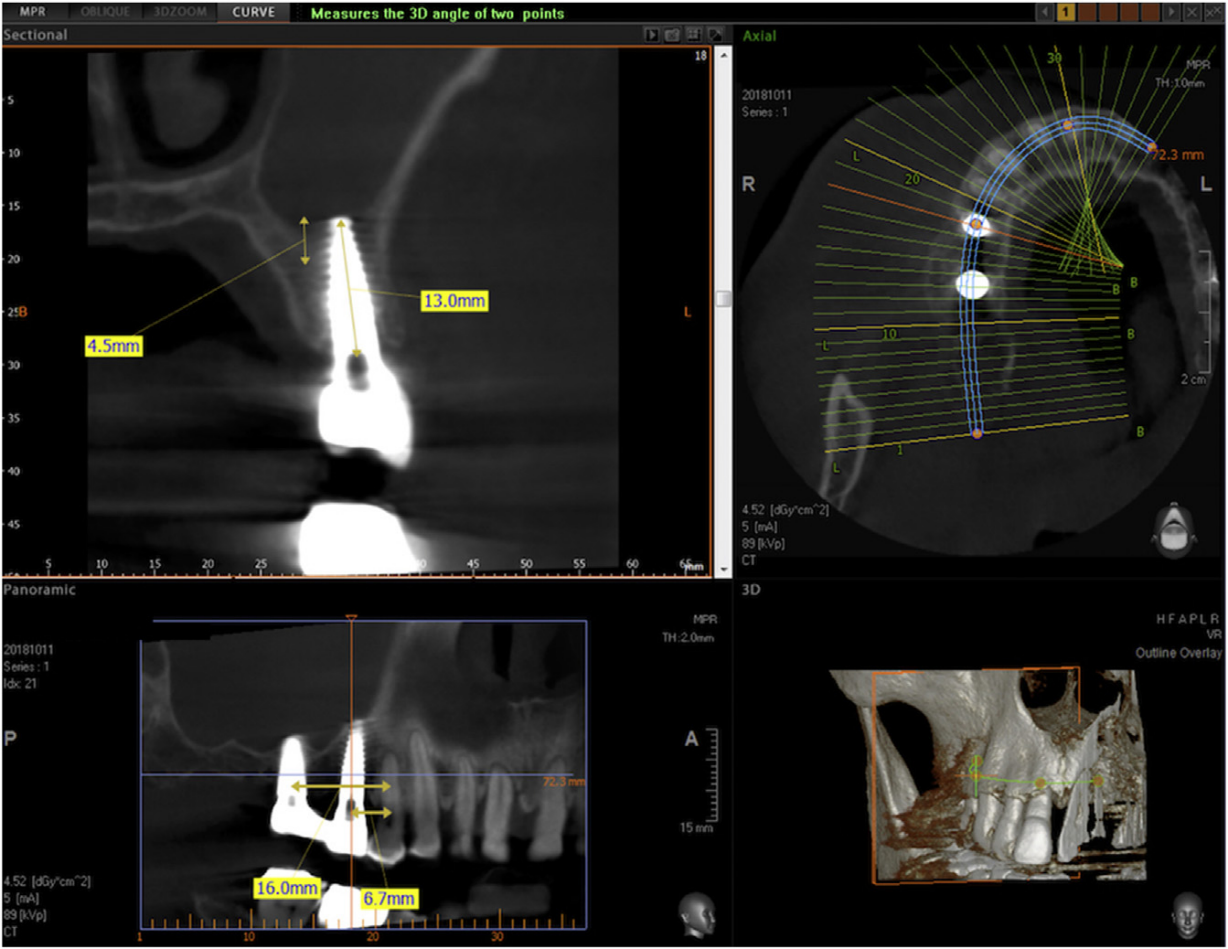
Detail of the CBCT of Figure 12 centered on the implant in the second bicuspid site. The implant is a 3.8 × 15 mm Sweden & Martina UXR. The apex of the implant is covered with newly formed bone. The vertical bone augmentation is about 4.5 mm.

In patients with chronic asymptomatic sinusitis, a sectional CBCT is scheduled one year after surgery. The last figures show the vertical bone gain measured on the preoperative and postoperative CBCT scans of one such patient (Figures 10, 11, 12, and 13) If sinusitis persisted the clinicians prescribed a mometasone furoate spray (Nasonex, MSD Italia S.r.l., Rome, Italy) for 6 months for 15 days per month. After this period, the operators checked the patients for the absence of nasal, facial and olfactory symptoms and hypogeusia or ageusia [22, 23, 24].

## 3 Results

All included cases were reviewed, and the summary statistics were analyzed.

Qualitative variables were described as absolute and percentage frequencies, while quantitative variables were summarized as mean, minimum, maximum, and standard deviation.

The 95% Confidence Intervals for the mean were calculated using a one sample T-test.The 95% Confidence Intervals for one proportion were calculated with exact Clopper-Pearson method.

The survival analysis were performed using -Meier method. A visual graph representation were provided.

All analyses were performed with SPSS software and P-value < 0.05 has been used as the threshold for statistical significance.

Over the 5-year period, the surgeons treated 52 consecutive patients with the Flapless LMSF Technique. 45,3 % of the subjects (n = 24) were men, while 54,7% of them (n = 29) were women with a mean age of 59 years (range, 32–77 years).

In total, the operators inserted 71 implants with the Flapless LMSF approach.

The patients were followed up for a minimum of 9 months, and up to 66 months (mean = 29.8 months).

**Figure 14:**
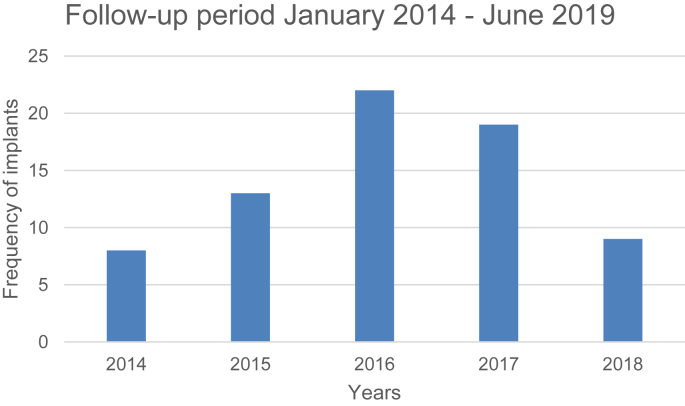
Follow-up period.

The follow-up period is shown in Figure 14.

87,3% of the implants (n = 62) were Sweden&Martina Global UXR with ZrTi surface and 12,7% of the implants (n = 9) were Straumann Bone Level Tapered (BLT) titanium with SLA surface.

The mean of implant width was 4,36 (95% CI 4,24 - 4,49).

The mean of implant length was 11,1 (95% CI 10,72 - 11,44).

Implant length, width, and type are listed in Table 1.

**Table 1:** Mean and relative 95% confidence interval.

Descriptive Statistics					
	N.	Minimum	Maximum	Mean	Std. Deviation
Bone height	71	4.0	7.5	5.059	.7003
Bone width	71	5.0	11.0	6.724	1.5185
Implant width	71	3.80	5.50	4.3606	.52678
Implant length	71	8.5	15.0	11.077	1.5181

	Mean	95% Confidence Interval	
		Lower	Upper
Bone height	5.0592	4.893	5.225
Bone width	6.7239	6.365	7.083
Implant width	4.36056	4.2359	4.4852
Implant length	11.0775	10.718	11.437

The sub-sinus bone height ranged between 4 and 7,5 mm with a mean of 5.06 mm (95% CI 4.89–5.23).

**Figure 15:**
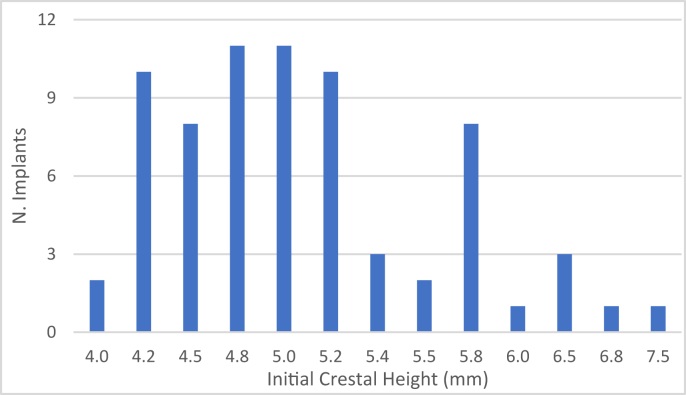
Number of implants placed according to initial residual crestal height.

The baseline height is shown in Figure 15.

The crestal width below the sinus ranged between 5 and 11 mm, with a mean of 6.72 mm (95% CI 6.37–7.08).

**Figure 16:**
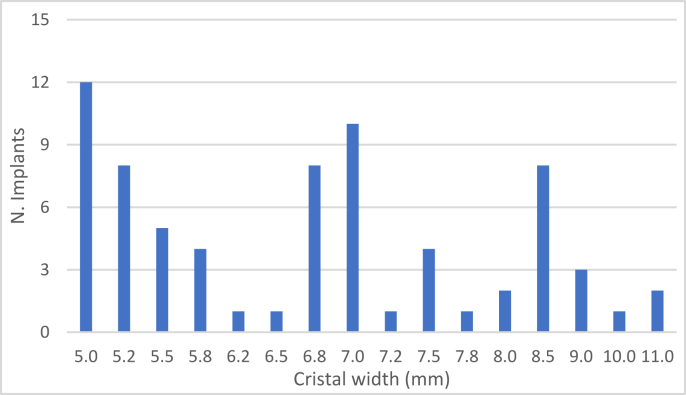
Number of implants placed according to initial crestal width.

The baseline crestal width is shown in Figure 16.

Implant positions in accordance with the FDI numbering system are listed in Table 2: 46,5% (n = 33) of the implants were placed in I quadrant, while the 53,5% (n = 38) of the implants were placed in the II quadrant.

**Table 2:** Distribution of implants.

Parameter	Frequency	Percent of total
Total	71	100
**Implants I quadrant**	**33**	**46,5%**
IMPLANT SITE 1.4	3	4,2%
IMPLANT SITE 1.5	8	11,3%
IMPLANT SITE 1.6	17	23,9%
IMPLANT SITE 1.7	5	7,0%
**Implants II quadrant**	**38**	**53,5%**
IMPLANT SITE 2.4	5	7,0%
IMPLANT SITE 2.5	11	15,5%
IMPLANT SITE 2.6	15	21,1%
IMPLANT SITE 2.7	7	9,8%

One implant failed during the osseointegration period and was successfully replaced after the healing period. This failure corresponds to 1.92% of the sample. The exact 95% Confidence Interval is 0.05%–10.26% [25].

All remaining implants were successfully loaded with the final fixed metal-ceramic or monolithic zirconia prosthesis and remained successfully in function during the follow-up period: 18 implants replaced single missing molars, and 53 implants replaced multiple missing posterior teeth.

No cases of complications or bleeding were encountered during the surgical procedure. In 32 sites, a collagen fleece was placed at the end of the osteotomy before implant insertion.

None of the cases developed postoperative hematoma of clinical relevance.

**Figure 17:**
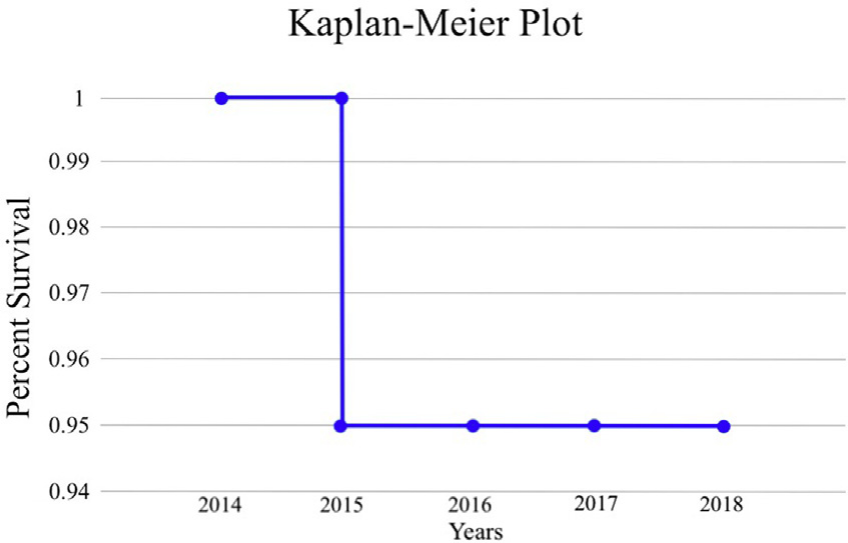
Kaplan-Meier plot.

The cumulative success rate of this ongoing multicenter prospective longitudinal clinical study during the 5-year observation period was 95% (Table 3, Figure 17).

**Table 3:** Survival analysis with the Kaplan-Meier method.

Interval	Years	Risk in the period	Events	Censored	Survival in the period	Cumulative Survival
1	0–1	8	0	0	1	1
2	1–2	13	0	0	1	1
3	2–3	22	1	0	0,95	0,95
4	3–4	19	0	0	1	0,95
5	4–5	9	0	0	1	0,95

## 4 Discussion

Within the limits of this study, the Flapless LMSF technique proved a reliable alternative to more traditional sinus lift and other osteotome mini-lift techniques. Of course, the Flapless LMSF must be applied exclusively to select cases where the band of keratinised tissue is sufficient and need not be augmented with flap procedures; in addition, the bone width of the ridge must also be sufficient for the planned implant without the need for breadth expansion.

If the keratinised tissue and/or the bone width is insufficient the classical LMSF technique can be used to obtain a horizontal expansion with a laterally and apically repositioned partial-thickness flap.

Accurate planning with preoperative clinical, radiographic and CBCT examination is fundamental to plan the correct treatment strategy and operate a careful case selection.

Patient selection is based almost exclusively on anatomical implant site considerations. Patient exclusion is limited to a very few general conditions.

When compared to the lateral window technique, the Flapless LMSF is minimally invasive and with faster healing times. On the other hand, the disadvantage is that it can be applied only in a limited number of select cases with the appropriate anatomical characteristics.

When compared to other osteotome or crestal access sinus lifts (such as the various hydraulic techniques), healing times are comparable.

An advantage of this technique, as is also the case of the traditional LMSF technique, is that only native bone is used for augmentation and there is no need for additional grafting.

All implants used in this study are “root-form”. It is the opinion of the authors that a more cylindrical design is inappropriate, since it would be more difficult to engage the PNR and to give directionality to the implant. In addition, the magnetic mallet osteotomes are conical and so more readily adaptable to root-form implants.

Although this technique is applicable only to select cases, it is the opinion of the authors that it is unnecessary to raise a flap in such cases, even when the height of the bone is in the 4mm range, since this technique is not more “blind” than a classical LMSF.

Very few similar flapless crestal sinus lift techniques have been described in the literature, using piezosurgery, balloon and CBCT guided Summer's Osteotome technique. All these techniques were deemed successful and with low morbidity by the respective authors [26, 27, 28].

## 5 Conclusions

Although the number of cases studied is limited, the findings of this 5-year study indicate that dental implants placed in the posterior maxilla in edentulous patients classifiable in Kennedy Classes I, II and III using the Flapless LMSF Technique shows excellent clinical success rates, within the limits of this relatively short follow-up period. Long-term clinical follow-up data is needed and will be published further on in this ongoing clinical study. It must be also considered that this technique is suitable only for select anatomical situations.

## Declarations

### Author contribution statement

Ernesto Bruschi: Conceived and designed the experiments; Performed the experiments; Analyzed and interpreted the data; Wrote the paper.

Giovanni B. Bruschi: Performed the experiments.

Laura Papetti: Analyzed and interpreted the data; Contributed reagents, materials, analysis tools or data; Wrote the paper.

### Funding statement

This research did not receive any specific grant from funding agencies in the public, commercial, or not-for-profit sectors.

### Data availability statement

The data that has been used is confidential.

### Declaration of interests statement

The authors declare no conflict of interest.

### Additional information

No additional information is available for this paper.

## References

[1] Tatum H. (1986). Maxillary and sinus implant reconstructions. Dent. Clin. North Am..

[2] Boyne P.J., James R.A. (1980). Grafting of the maxillary sinus door with autogenous marrow and bone. J. Oral Surg..

[3] Jensen T., Schou S., Stavropoulos A., Terheyden H., Holmstrup P. (2012). Maxillary sinus floor augmentation with Bio-Oss or Bio-Oss mixed with autogenous bone as graft: a systematic review. Clin. Oral Implants Res..

[4] Aghaloo T.L., Misch C., Lin G.H., Iacono V.J., Wang H.L. (2016). Bone augmentation of the edentulous maxilla for implant placement: a systematic review. Int. J. Oral Maxillofac. Implants.

[5] Danesh-Sani S.A., Engebretson S.P., Janal M.N. (2017). Histomorphometric results of different grafting materials and effect of healing time on bone maturation after sinus floor augmentation: a systematic review and meta-analysis. J. Periodontal. Res..

[6] Starch-Jensen T., Aludden H., Hallman M., Dahlin C., Christensen A.E., Mordenfeld A. (2018). A systematic review and meta-analysis of long-term studies (five or more years) assessing maxillary sinus floor augmentation. Int. J. Oral Maxillofac. Surg..

[7] Ting M., Rice J.G., Braid S.M., Lee C., Suzuki J.B. (2017). Maxillary sinus augmentation for dental implant rehabilitation of the Edentulous Ridge: a comprehensive overview of systematic reviews. Implant Dent..

[8] Summers R.B. (1998). Sinus floor elevation with osteotomes. J. Esthetic Dent..

[9] Rosen P.S., Summers R., Mellado J.R., Salkin L.M., Shanaman R.H., Marks M.H., Fugazzotto P.A. (1999). The bone-added osteotome sinus floor elevation technique: multicenter retrospective report of consecutively treated patients. Int. J. Oral Maxillofac. Implants.

[10] Better H., Slavescu D., Barbu H., Cochran D.L., Chaushu G. (2014). Minimally invasive sinus lift implant device: a multicenter safety and efficacy trial preliminary results. Clin. Implant Dent. Relat. Res..

[11] Kao D.W., DeHaven H.A. (2011). Controlled hydrostatic sinus elevation: a novel method of elevating the sinus membrane. Implant Dent..

[12] Bruschi G.B., Scipioni A., Calesini G., Bruschi E. (1998). Localized management of sinus floor with simultaneous implant placement: a clinical report. Int. J. Oral Maxillofac. Implants.

[13] Chan H.L., Monje A., Suarez F., Benavides E., Wang H.L. (2013). Palatonasal recess on medial wall of maxillary sinus and clinical implications for sinus augmentation via lateral window approach. J. Periodontol..

[14] Scipioni A., Bruschi G.B., Calesini G. (1994). The edentulous ridge expansion technique: a five-year study. Int. J. Periodontics Restor. Dent..

[15] Scipioni A., Bruschi G.B., Giargia M., Berglundh T., Lindhe J. (1997). Healing at implants with and without primary bone contact. Clin. Oral Implants Res..

[16] Beolchini M., Lang N.P., Viganò P., Bengazi F., Triana B.G., Botticelli D. (2014 Oct). The edentulous ridge expansion (ERE) technique an experimental study in the dog. Clin. Oral Implants Res..

[17] Cavicchia F., Bravi F., Petrelli G. (2001). Localized augmentation of the maxillary sinus floor through a coronal approach for the placement of implants. Int. J. Periodontics Restor. Dent..

[18] Winter A.A., Pollack A.S., Odrich R.B. (2002). Placement of implants in the severely atrophic posterior maxilla using localized management of the sinus floor: a preliminary study. Int. J. Oral Maxillofac. Implants.

[19] Bruschi G.B., Crespi R., Capparè P., Bravi F., Bruschi E., Gherlone E. (2013). Localized management of sinus floor technique for implant placement in fresh molar sockets. Clin. Implant Dent. Relat. Res..

[20] Crespi R., Capparè P., Gherlone E. (2013). Electrical mallet provides essential advantages in maxillary bone condensing. A prospective clinical study. Clin. Implant Dent. Relat. Res..

[21] (2013). World medical association declaration of Helsinki. J. Am. Med. Assoc..

[22] Mösges R., Bachert C., Rudack C., Hauswald B., Klimek L., Spaeth J., Rasp G., Vent J., Hörmann K. (2011). Efficacy and safety of mometasone furoate nasal spray in the treatment of chronic rhinosinusitis. Adv. Ther..

[23] Passali D., Spinosi M.C., Crisanti A., Bellussi L.M. (2016). Mometasone furoate nasal spray: a systematic review. Multidiscip. Respir. Med..

[24] Rudmik L., Soler Z.M. (2015). Medical therapies for adult chronic sinusitis: a systematic review. J. Am. Med. Assoc..

[25] Newcombe R.G. (1998). Two-sided confidence intervals for the single proportion: comparison of seven methods. Stat. Med..

[26] Mazor Z., Kfir E., Lorean A., Mijiritsky E., Horowitz R.A. (2011). Flapless approach to maxillary sinus augmentation using minimally invasive antral membrane balloon elevation. Implant Dent..

[27] Fornell J., Johansson L.Å., Bolin A., Isaksson S., Flapless Sennerby L. (2012 Jan). CBCT-guided osteotome sinus floor elevation with simultaneous implant installation. I: radiographic examination and surgical technique. A prospective 1-year follow-up. Clin. Oral Implants Res..

[28] Kim J.M., Sohn D.S., Bae M.S., Moon J.W., Lee J.H., Park I.S. (2014). Flapless transcrestal sinus augmentation using hydrodynamic piezoelectric internal sinus elevation with autologous concentrated growth factors alone. Implant Dent..

